# The occurrence of antimicrobial resistance and class 1 integrons among commensal *Escherichia coli *isolates from infants and elderly persons

**DOI:** 10.1186/1476-0711-8-34

**Published:** 2009-12-08

**Authors:** Epp Sepp, Jelena Stsepetova, Krista Lõivukene, Kai Truusalu, Siiri Kõljalg, Paul Naaber, Marika Mikelsaar

**Affiliations:** 1 Department of Microbiology, University of Tartu, Ravila 19, 50411 Tartu, Estonia; 2 United Laboratories, Tartu University Hospitals, Puusepa 1A, 50406 Tartu, Estonia; 3 Department of Medical Microbiology, Stavanger University Hospital, Postboks 8100, 4068 Stavanger, Norway

## Abstract

**Background:**

The aim of our study was to compare the presence of the *intI1 *gene and its associations with the antibiotic resistance of commensal *Escherichia coli *strains in children with/without previous antibiotic treatments and elderly hospitalized/healthy individuals.

**Methods:**

One-hundred-and-fifteen intestinal *E. coli *strains were analyzed: 30 strains from 10 antibiotic-naive infants; 27 from 9 antibiotic-treated outpatient infants; 30 from 9 healthy elderly volunteers; and 28 from 9 hospitalized elderly patients. The MIC values of ampicillin, cefuroxime, cefotaxime, gentamicin, ciprofloxacin, and sulfamethoxazole were measured by E-test and *IntI1 *was detected by PCR.

**Results:**

Out of the 115 strains, 56 (49%) carried class 1 integron genes. Comparing persons without medical interventions, we found in antibiotic-naive children a significantly higher frequency of integron-bearing strains and MIC values than in healthy elderly persons (53% versus 17%; p < 0.01). Evaluating medical interventions, we found a higher resistance and frequency of integrons in strains from hospitalized elderly persons compared with non-hospitalized ones. Children treated with antibiotics had strains with higher MIC values (when compared with antibiotic-naive ones), but the integron-bearing in strains was similar. In most cases, the differences in resistance between the groups (integron-positive and negative strains separately) were higher than the differences between integron-positive and negative strains within the groups.

**Conclusion:**

The prevalence of integrons in commensal *E. coli *strains in persons without previous medical intervention depended on age. The resistance of integron-carrying and non-carrying strains is more dependent on influencing factors (hospitalization and antibiotic administration) in particular groups than merely the presence or absence of integrons.

## Background

*Escherichia coli *is one of the most common bacteria in the environment and in the intestinal tract of humans and animals [1-3]. The intensive use of antibiotics in both human and veterinary medicine, as well as in the field of agriculture, is associated with an emerging resistance against therapeutic drugs, followed by the selection of virulence and resistance gene cassettes (such as integrons) carrying *E. coli *strains in humans, animals, and the environment [4-6].

Integrons are ancient structures that contain determinants of a site-specific recombination system to capture genes encoding antimicrobial resistance [7]. They can locate within transposons or conjugative plasmids and contribute to the gene traffic leading to the acquisition of new genes in bacteria [8-13].

The class 1 integron is prevalent on plasmids. It has been found in both gram-positive and especially in gram-negative bacteria like *Enterobacteriaceae, Campylobacter *spp., *Acinetobacter *spp., *Pseudomonas *spp., *Aeromonas *spp., and *Vibrio *spp. [13-16]. The prevalence of the class 1 integron in clinical *E. coli *strains is 33 to 49% [10,17-20].

Class 1 integrons carrying commensal *E. coli *strains have been isolated from 11%-42% of healthy adult persons [17,19,21]. The high prevalence of the *intI1 *gene among the intestinal *E. coli *suggests that commensals could represent an important reservoir of resistance determinants, where gene cassettes could spread between commensal and pathogenic bacteria [1].

Still, there are few data assessing the association between integron carriage and the antimicrobial resistance patterns of *E. coli *isolated from the commensal gut microbiota of different age groups like infants and elderly persons [17,19,21,22].

We aimed to compare the presence of the *intI1 *gene and its association with the antibiotic resistance of commensal *E. coli *strains in patients of different age groups: children with/without previous antibiotic treatment and elderly hospitalized/healthy individuals.

## Methods

### Subjects

Four groups of subjects were included into the study: (Group 1) antibiotic-naive infants (n = 10; age 3-12 months; mean 7.5 months); (Group 2) antibiotic-treated outpatient infants (n = 9; age 1 week-12 months; mean 7.4 months); (Group 3) healthy elderly volunteers (n = 9; age 68-80 years; mean 73 years), and; (Group 4) elderly hospitalized patients (n = 9; age 65-77 years; mean 70 years). All children were vaginally born at the Women's Clinic of Tartu University Hospital and were randomly selected from the participants of a prospective study on the bias of data concerning previous antibiotic treatment [23]. The healthy volunteers, randomly selected from a registry of general practitioners in Tartu, were considered to be healthy, had not been following any special dietary routines and had not received antibiotic treatment for at least two months prior to inclusion. The hospitalized elderly, without clinical signs indicating inflammatory disease, were randomly recruited from the Department of Orthopaedics of Tartu University Hospital two to three days before scheduled orthopaedic surgery and had not received antibiotics during the previous two months.

### Faecal samples

From 27 persons, approximately 1 gram of freshly voided stool was collected into sterile plastic containers and frozen at -20°C. Weighed samples of faeces were serially diluted (10^-2^-10^-9^) in a pre-reduced phosphate buffer (pH 7.2). A quantitative analysis of the gut bacteria was performed using seeding duplicate samples of 0.1 ml of each dilution on MacConkey (Oxoid, England) agar plates. The colony counts of the different faecal dilutions were recorded, and all colonies of different morphology from the highest dilutions with growth were isolated and identified with the standard methods. From each sample (n = 27), 3-4 fenotypically different dominating *E. coli *isolates were included. In total, 115 isolates were collected and analyzed.

### Antibiotic resistance

Antimicrobial susceptibility testing was performed using the E-test (AB Biodisk, Solna, Sweden) on Mueller-Hinton agar (Oxoid, UK). The values of the minimal inhibitory concentration (MIC) of *E. coli *isolates to ampicillin, cefuroxime, cefotaxime, gentamicin, ciprofloxacin, and sulfamethoxazole were determined according to the manufacturer's instructions and to Clinical and Laboratory Standard Institute guidelines.

### Detection of the class 1 integrons

All *E. coli *isolates were tested for the presence of class 1 integrons with a polymerase chain reaction (PCR) amplification of a class 1 integrase-specific fragment of the *intI1 *gene. The total DNA of the *E. coli *was extracted using a QIAamp DNA Mini Kit (Qiagen, Germany) following the manufacturer's protocol for gram-negative bacteria. The primer sequences used were 5'CS: 5'-GGCATCCAAGCAGCAAG-3' and 3'CS: 5'-AAGCAGACTTGACCTGA-3' [24]. The reactions were carried out in a volume of 50-μl containing 10× PCR buffer, 2.5 mM MgCl_2_, 2.5 mM dNTPs, 5 pmol/L each primer, 2.5 U Taq polymerase (Fermentas, Lithuania), and 1 μg of template DNA was added. Amplification specifications were as follows: 94°C for 5 min. followed by 35 cycles of 94°C for 30 sec., 55°C for 30 sec., 72°C for 30 sec. and the final extension at 72°C for 7 min. The amplicons were electrophorezed in 1.0% agarose gel and the 1 kb ladder (Fermentas, Lithuania) was used as a molecular size marker.

### Statistical analysis

The statistical analyses were performed using the SigmaStat (Jandel Scientific, USA) and Excel (Microsoft Corp.) software programs, employing the Fisher exact test, the chi-square test, and the Mann-Whitney rank sum test. P values less than 0.05 were considered statistically significant.

### Approval by ethics committee

The Ethics Committee of Tartu University Medical Faculty approved the study (No. 139/16 20.06.2005 and No. 152/52 18.09.2006).

## Results

### Prevalence of class 1 integrons in *E. coli *isolates in different study groups

Out of the 115 dominating commensal *E. coli *isolates, 56 (49%) carried the *intI1 *gene. We found a similarly high rate of integrons among dominating *E. coli *isolates in antibiotic-treated (Group 2; 17/27; 63%) and non-treated (Group 1; 16/30; 53%) infants, as well as in hospitalized elderly patients (Group 4; 18/28; 64%). In elderly healthy volunteers (Group 3), the integron-positive isolates were comparatively low among the dominating *E. coli *population (5/30; 17%; p < 0.01; Table 1).

**Table 1:** Prevalence of class 1 integrons among studied persons and *E. coli *isolates

	Infants		Elderly	
	
	Group1	Group2	Group3	Group4
**Persons with integrons/total (%)**	7/10 (70%)	8/9 (89%)	3/9 (33%)	8/9 (89%)

**Isolates with integrons/total (%)**	16/30 (53%)	17/27 (63%)	5/30 (17%)	18/28 (64%)

P values for comparisons between groups have been shown in the text.

### Antibiotic susceptibility of *E. coli *isolates

Comparing different age groups not influenced by medical intervention, we found that dominating *E. coli *isolates originating from antibiotic-naive infants (Group1) had higher MIC values to sulfamethoxazole (p < 0.001), cefotaxime (p = 0.003), gentamicin (p < 0.001), and ciprofloxacin (p < 0.001) than those originating from healthy elderly persons (Group3).

To evaluate the influence of medical interventions on dominating *E. coli *populations, we compared the MIC values of isolates of antibiotic-naive versus treated infants and healthy elderly persons versus hospitalized. The isolates from antibiotic-treated infants (Group2) were more resistant to different antibiotics (range 0-5, median 2 versus range 0-3, median 1; p = 0.025) and had higher MIC values to sulfamethoxazole (p = 0.048), cefuroxime (p < 0.001), cefotaxime (p = 0.013), gentamicin (p = 0.003), and ciprofloxacin (p = 0.002), compared to antibiotic-naive infants (Group1). In isolates from hospitalized patients (Group 4), the resistance to tested antibiotics (range 0-6, median 1 versus range 0-2 median 0; p = 0.01) and MIC values were higher to sulfamethoxazole (p < 0.001), ampicillin (p < 0.001), cefuroxime (p < 0.001), cefotaxime (p < 0.001), and ciprofloxacin (p < 0.001), when compared to their outpatient counterparts (Group 3).

### Relation between the presence of the *intI1 *gene and antibiotic susceptibility

**Figure 1:**
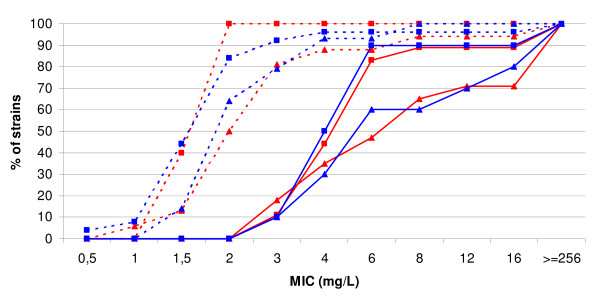
**Cumulative MIC values of cefuroxime**. red lines/marks - integron-positive strains; blue lines/marks - integron-negative strains; triangles - strains of children; squares - strains of adults; dotted line - strains of uninfluenced persons (antibiotic-naive children or healthy elderly); continuous line - strains of persons with medical interventions (antibiotic-treated children or hospitalized elderly).

In comparing strains from all the groups together, the MIC-s of *intI1*-positive isolates were significantly higher than integron-negative ones in the case of several antibiotics (sulfamethoxazole, p = 0.001; ampicillin, p < 0.001; cefuroxime, p = 0.003; cefotaxime, p < 0.001; ciprofloxacin, p = 0.038). However, in comparing the MIC values of *intI1*-positive and negative isolates in particular groups, we found a significant difference only in non-treated infants (Group 1) in the case of cefotaxime (p = 0.023). Comparing only *intI1*-negative isolates, we found significantly higher MIC values in groups with medical interventions (i.e. antibiotic-treated infants and hospitalized patients versus non-treated infants and non-hospitalized patients). Similar trends were also evident if only *intI1*-positive isolates were compared (Table 2). Thus, differences in MIC values were related to age in groups without medical interventions (infants versus elderly) and with medical interventions (antibiotic treatment and hospitalization), but not to the carriage or absence of integrons within a particular group. Figure 1 shows the cumulative MIC values of different groups in the case of cefuroxime.

**Table 2:** MIC_50 _values of intestinal dominant *E. coli *population in studied groups

Antibiotics	MIC_50 _(mg/L) of all isolates MIC_50 _of integron-negative/integron-positive isolates				Statistical significance*
		
	Children		Elderly		
		
	Group 1	Group 2	Group 3	Group 4	
**Ampicillin**	5 3^1^/7	6 10^1^/6	2 2^2^/256	6 6^2^/36	^1 ^p = 0.05 ^2 ^p = 0.001

**Cefuroxime**	2 2^1^/2.5^2^	6 6^1^/8^2^	2 2^3^/2^4^	6 5^3^/6^4^	^1 ^p < 0.001 ^2 ^p < 0.001 ^3 ^p < 0.001 ^4 ^p < 0.001

**Cefotaxime**	0.064 0.032^1^/0.094	0.125 0.125^1^/0.125	0.047 0.047^2^/0.047^3^	0.125 0.125^2^/0.125^3^	^1 ^p = 0.028 ^2 ^p < 0.001 ^3 ^p = 0.001

**Gentamicin**	0.38 0.38/0.44^1^	1.5 1.0/1.5^1^	0.5 0.5/0.38	0.38 0.38/0.38	^1 ^p = 0.011

**Ciprofloxacin**	0.012 0.012/0.012^1^	0.023 0.023/0.023^1^	0.008 0.008^2^/0.012^3^	0.016 0.016^2^/0.016^3^	^1 ^p = 0.004 ^2 ^p < 0.001 ^3 ^p = 0.048

**Sulfamethoxazole**	512 20/512	512 512/512	24 24^1^/512	64 228^1^/56	^1 ^p = 0.03

*Statistical significance (by the Mann-Whitney test) has been shown separately for integron-positive and negative strains comparing groups with medical intervention to groups without intervention (i.e. antibiotic-naive children versus treated ones; hospitalized elderly versus non-hospitalized). P values for the comparison of the other groups have been shown in the text.

## Discussion

We found a significantly higher frequency of the *intI1 *gene in the dominating intestinal *E. coli *populations in healthy antibiotic-naive children (Group 1; 53%) compared to healthy elderly persons (Group3; 17%). The MIC values for several antibiotics for dominant *E. coli *isolates were lower in elderly persons than in children. Although some previous studies have shown variations of integron prevalence in *E. coli *isolates from the intestinal tracts of healthy persons, no significant differences in different age groups (not influenced by antibiotics and hospitalization) in the same region has been described yet [5,21,22,25].

Our finding is somewhat surprising, since one can assume a higher rate of resistant and integron-containing bacteria in older people due to the selective pressure of antibiotics consumed during a lifespan. The possible explanation is that in young children (<1 years), the gastrointestinal micro-ecosystem is not yet fully completed and colonization resistance (supported by anaerobes, lactobacilli, and other endogenous commensal bacteria) against exogenous bacteria is weaker; hence, they may be colonized by high numbers of *E. coli *strains from the environment, containing different virulence or resistance genes, e.g. integrons. Furthermore, during the development of commensal intestinal microflora, the strains without resistance probably have advantages in microbial competition in the intestinal micro-ecosystem, and due to the fitness cost the resistant population would be suppressed (in the case of the absence of an antibiotic pressure) [26].

Comparing the dominating *E. coli *populations of antibiotic-naive and antibiotic-influenced infants, we found a similar high rate in the presence of class 1 integrons. However, the resistance of *E. coli *was significantly higher in antibiotic-treated infants. Our results indicate that the higher resistance in antibiotic-treated infants is probably mediated by integron-independent mechanisms, or the expression of resistance genes in integrons is due to antibiotic consumption.

We found that the dominating *E. coli *population in the intestinal tracts of hospitalized elderly patients was more resistant and more frequently harbored integrons than did those in healthy persons. However, this resistance was not mainly integron-mediated, since similar differences in MIC values were found when separately comparing integron-positive and negative isolates. The published data concerning the prevalence of integrons in *E. coli *strains isolated from a hospital and community are contradictory. Some investigators have found that higher numbers of integron-positive isolates originated from patients of intensive care units, when compared to non-intensive care unit ones and outpatients (82% versus 10% and 8%) [25]. To the contrary, Lee and co-workers indicated that the prevalence of class 1 integrons among commensal *E. coli *isolates was similar to that of clinical *E. coli *isolates from hospital-acquired infections [27].

Increasing resistance in the dominating populations of intestinal bacteria could be caused by the acquisition of resistant and/or integron-positive strains from the hospital environment or by the overgrowth of normally suppressed resistant populations. In both cases, the disruption of commensal microflora responsible for the maintenance of colonization resistance is a conducive factor. This can happen due to a recent exposure to antibiotics, a different diet, increased hygiene habits, and a stressful situation, in essence describing a hospital environment [23,26,28]. It has been previously indicated that exposure to stress results in a significant alteration of intestinal microflora: the counts of beneficial bacteria decrease and enterobacteria like *E. coli *increase [29,30].

In our study, all integron-positive isolates together had higher MIC values to antibiotics than integron-negative isolates, but susceptibility was similar in studied particular groups. In previous studies, resistant phenotypes usually correlated with the presence of integrons [25,31,32]. Several studies have described the association between the presence of class 1 integrons and non-susceptibility to sulfamethoxazole, β-lactams (ampicillin, piperacillin, cefuroxime), aminoglycosides (gentamicin, tobramycin), and fluoroquinolone (ciprofloxacin) in *E. coli *strains [20,24]. It has been supposed that the screening of integrons could assist in guiding treatment regimens and could complement existing antibiotic resistance surveillance programs by providing information about both aspects: resistance and its dissemination between commensal and infectious strains [32]. On the one hand, the absence of significant differences in resistance between integron-positive and negative isolates within particular groups in our study may be explained with the small number of strains/persons. However, on the other hand, significant differences in resistance between these groups (separately comparing integron-positive and negative isolates) indicate that the presence of integrons is not important for the predisposition or correlation of the general antimicrobial resistance. Thus, other factors are more important in determining the resistance of the predominant population of *E. coli *and probably other facultative pathogens as well. For a more complete understanding of these results, further studies detecting particular resistance genes and the presence of empty integrons are needed.

## Conclusion

In our study, the prevalence of the *intl1 *gene in commensal *E. coli *isolates differs between age groups and depends on hospitalization. Resistance (to the antibiotics selected in our study) of *intl1*-carrying and non-carrying isolates is more dependent on the influencing factors (such as hospitalization or antibiotic administration) on particular groups than merely the presence or absence of integrons.

## Competing interests

The authors declare that they have no competing interests.

## Authors' contributions

ES - study design, drafting the manuscript; PN - statistics and data interpretation, revising the manuscript; JS - molecular studies; SK - antimicrobial resistance testing, drafting the manuscript; KL - collection of the samples and clinical data, interpretation of data; MM - study design, revision of the manuscript; KT - microbiological studies, final correction of the manuscript. All authors have read and approved the final version of manuscript.
